# Supplementary material for: Development of a Single Molecule Counting Assay to Differentiate Chromophobe Renal Cancer and Oncocytoma in Clinics
Source: Cancers (Basel). 2022 Jul 1;14(13):3242. doi: 10.3390/cancers14133242 (PMC9265083; doi:10.3390/cancers14133242)
Supplement: Supplementary file 1 [file cancers-14-03242-s001.zip › cancers-1743129-supplementary.pdf]

Supplementary Material: Development of a Single Molecule Counting Assay to Differentiate Chromophobe Renal Cancer and Oncocytoma in Clinics

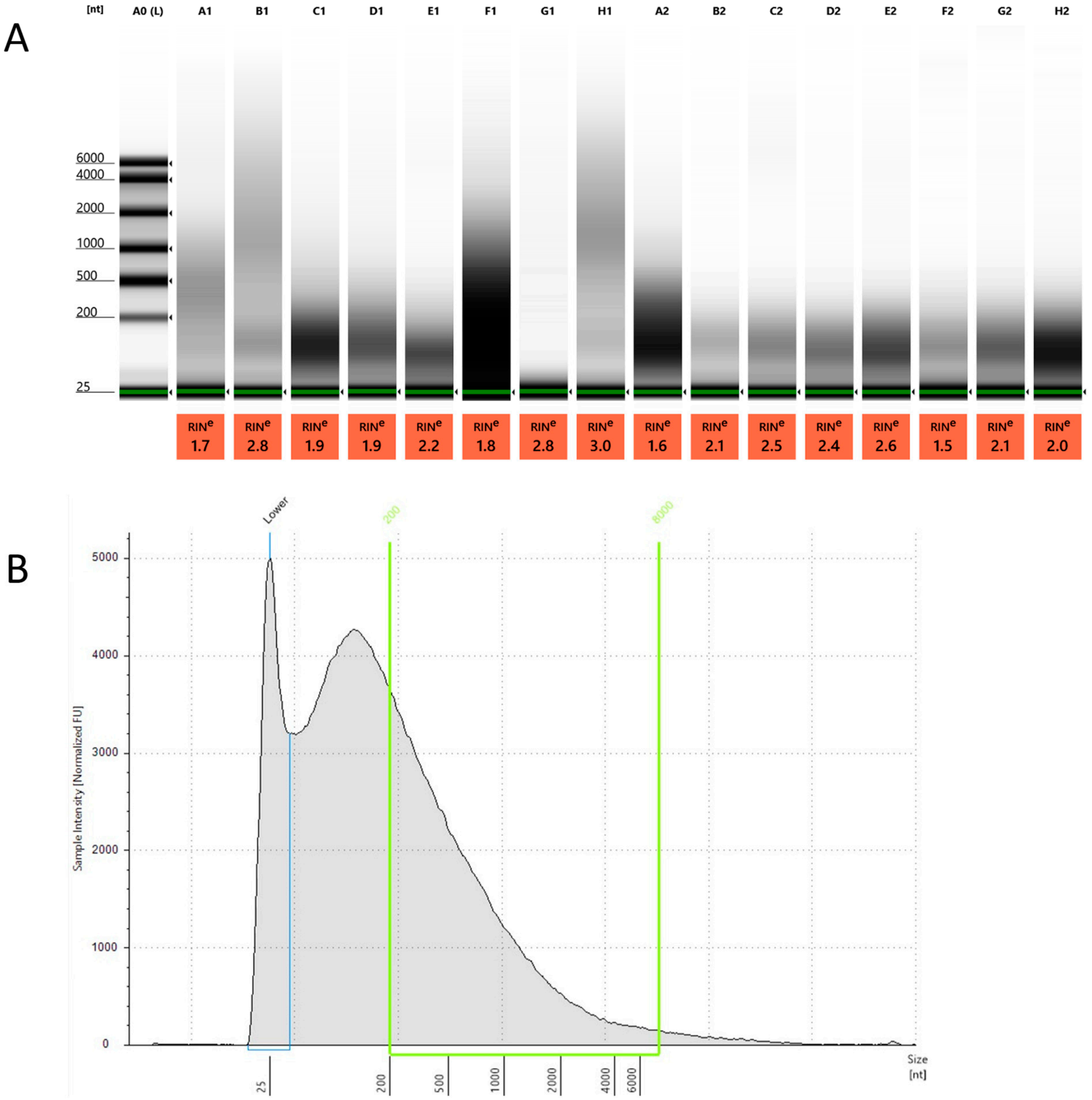

Figure S1. RNA quality control on Tapestation 2200. **A**: Gel images showing RNA quality. The bottom number represents RIN (RNA integrity number). **B**: Showing a representative sample for RNA fragments. The green horizontal line represents the target range for hybridization. The RNA concentration was calculated for this range for each sample.

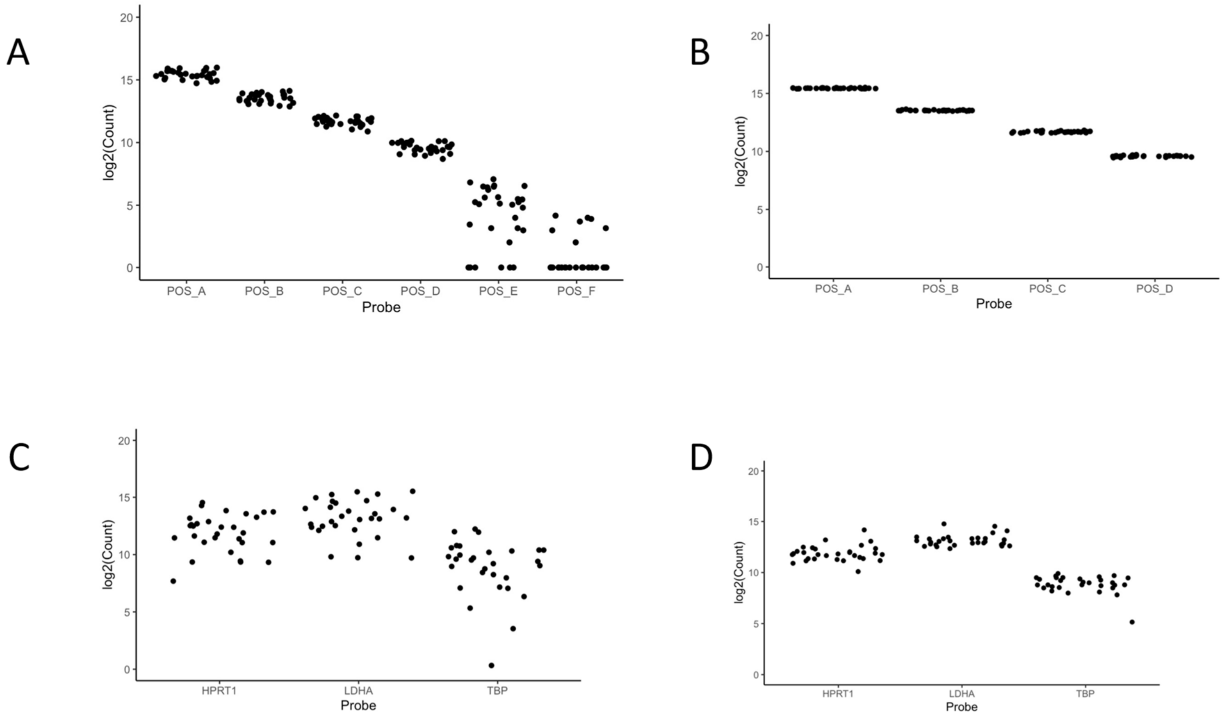

Figure S2. Positive control and housekeeping gene normalization using geometric mean. **A**: Before normalization of the positive control **B**: After positive control normalization. **C**: Before housekeeping gene normalization. **D**: After housekeeping gene normalization.

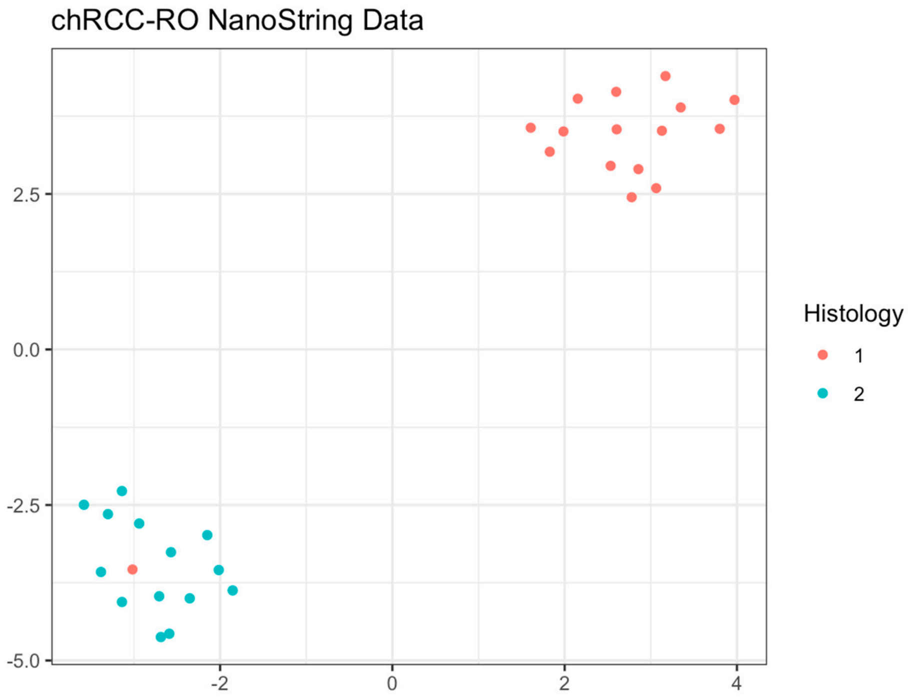

Figure S3. Uniform manifold approximation and projection (UMAP) analysis showing two clusters. Raw count data was normalized with internal controls and log2 transformed prior to UMAP analysis. Histology 1 represents chRCC and 2 represents RO samples. .

Table S1. Comparison of original hematoxylin-eosin stained sections at the time of diagnosis with a re-review of the slides by three independent pathologists. The identity and diagnosis were blinded to be reviewed by pathologists.

Sample ID	Histology	Reviewer 1	Reviewer 2	Reviewer 3
Rand1	chRCC	chRCC	chRCC	chRCC
Rand10	chRCC	chRCC	chRCC	chRCC
Rand11	chRCC	chRCC	chRCC	chRCC
Rand12	RO	RO	RO	RO
Rand13	chRCC	chRCC	chRCC	chRCC
Rand14	chRCC	chRCC	chRCC	chRCC
Rand15	RO	RO	RO	RO
Rand16	chRCC	chRCC	chRCC	chRCC
Rand17	RO	RO	RO	RO
Rand18	chRCC	chRCC	chRCC	chRCC
Rand19	RO	RO	RO	RO
Rand2	chRCC	chRCC	chRCC	chRCC
Rand20	chRCC	chRCC	chRCC	chRCC
Rand21	RO	RO	RO	RO
Rand22	RO	RO	RO	RO
Rand23	RO	RO	RO	RO
Rand24	RO	RO	RO	RO
Rand25	chRCC	chRCC	chRCC	chRCC
Rand26	chRCC	chRCC	chRCC	chRCC
Rand27	chRCC	chRCC	chRCC	chRCC
Rand28	RO	RO	RO	RO
Rand29	chRCC	chRCC	chRCC	chRCC
Rand3	chRCC	chRCC	chRCC	RO
Rand30	chRCC	chRCC	chRCC	chRCC
Rand31	chRCC	RO	RO	RO
Rand32	RO	RO	RO	RO
Rand4	chRCC	chRCC	chRCC	chRCC
Rand5	RO	RO	RO	RO
Rand6	RO	RO	RO	RO
Rand7	RO	RO	RO	RO
Rand8	RO	RO	RO	RO
Rand9	RO	RO	RO	RO

Table S2. Functional description for the COGS (Chromophobe-Oncocytoma Gene Signature) genes.

Gene ID	Description	Changes in chRCC
AP1M2	adaptor related protein complex 1 subunit mu 2	Upregulated
AQP6	aquaporin 6	Downregulated
ATP2C1	ATPase secretory pathway Ca2+ transporting 1	Upregulated
BSPRY	B-box and SPRY domain containing	Upregulated
CLDN8	claudin 8	Upregulated
DNAI3	dynein axonemal intermediate chain 3	Downregulated
ELMO3	engulfment and cell motility 3	Upregulated
ESRP1	epithelial splicing regulatory protein 1	Upregulated
HOOK2	hook microtubule tethering protein 2	Upregulated
ITGB3	integrin subunit beta 3	Downregulated
KCNG3	potassium voltage-gated channel modifier subfamily G member 3	Downregulated
KIDINS220	kinase D interacting substrate 220	Downregulated
KRT7	keratin 7	Upregulated
LAMA1	laminin subunit alpha 1	Upregulated
LIMS1	LIM zinc finger domain containing 1	Downregulated
LRFN5	leucine rich repeat and fibronectin type III domain containing 5	Upregulated
LSR	lipolysis stimulated lipoprotein receptor	Upregulated
MANEA	mannosidase endo-alpha	Downregulated
MAP4K3	mitogen-activated protein kinase kinase kinase kinase 3	Upregulated
MSH2	mutS homolog 2	Downregulated

NDUFS1	NADH:ubiquinone oxidoreductase core subunit S1	Downregulated
PLCL1	phospholipase C like 1 (inactive)	Downregulated
PLCL2	phospholipase C like 2	Upregulated
PNPT1	polyribonucleotide nucleotidyltransferase 1	Downregulated
PRDX3	peroxiredoxin 3	Downregulated
RSPO3	R-spondin 3	Upregulated
S100A1	S100 calcium binding protein A1	Downregulated
SOCS1	suppressor of cytokine signaling 1	Upregulated
SPINT2	serine peptidase inhibitor, Kunitz type 2	Upregulated
SUCLA2	succinate-CoA ligase ADP-forming subunit beta	Downregulated

Software and packages:

All statistical analyses were performed using the R language and environment for statistical computing (v4.1.2; R Foundation for Statistical Computing).

1. Quality control: NanoString nCounter output (RCC – reporter code count) was read by nSolver [1] for quality control for imaging QC, binding density, positive spike control and limit of detection QCs.

2. Determination of cutpoint: Optimum cut points were determined by cutpointr [2].

3. AUC analysis: AUC values between the tumor types were analyzed by catools [3].

4. Unsupervised learning: Unsupervised learning was implemented from UMAP [4] and Hierarchical clustering by ComplexHeatmap [5]. UMAP components were used as "features". Hierarchical Clustering was implemented from Complex heatmap (clustering distance = "maximum" and clustering method = "ward.D").

5. Supervised learning: Supervised learning models were Random forest, support vector machine and generalized linear model implemented from Caret [6] package. Supervised UMAP was implemented from tidymodels [7].

6. Linear Mixed model: Linear mixed models were implemented from lme4 [8].

All codes and data are available at GitHub (https://github.com/kbsatter/chRCC-paper-2).

Reference

1. NSolver Advanced Analysis Software Available online: https://nanostring.com/products/analysis-solutions/nsolver-advanced-analysis-software/ (accessed on 9 May 2022).
2. Thiele, C. *Cutpointr: Determine and Evaluate Optimal Cutpoints in Binary Classification Tasks*; 2021;
3. Tuszynski, J. *CaTools: Tools: Moving Window Statistics, GIF, Base64, ROC AUC, Etc*; 2021;
4. McInnes, L.; Healy, J.; Melville, J. UMAP: Uniform Manifold Approximation and Projection for Dimension Reduction. *arXiv:1802.03426 [cs, stat]* **2020**.
5. Gu, Z. *ComplexHeatmap: Make Complex Heatmaps*; Bioconductor version: Release (3.12), 2021;
6. Kuhn, M.; Wing, J.; Weston, S.; Williams, A.; Keefer, C.; Engelhardt, A.; Cooper, T.; Mayer, Z.; Kenkel, B.; R Core Team; et al. *Caret: Classification and Regression Training*; 2020;
7. Tidymodels Available online: https://www.tidymodels.org/ (accessed on 29 April 2022).
8. Bates, D.; Mächler, M.; Bolker, B.; Walker, S. Fitting Linear Mixed-Effects Models Using Lme4. *Journal of Statistical Software* **2015**, *67*, 1–48, doi:10.18637/jss.v067.i01.
